# 3D Printing of NiCoP/Ti_3_C_2_ MXene Architectures for Energy Storage Devices with High Areal and Volumetric Energy Density

**DOI:** 10.1007/s40820-020-00483-5

**Published:** 2020-07-09

**Authors:** Lianghao Yu, Weiping Li, Chaohui Wei, Qifeng Yang, Yuanlong Shao, Jingyu Sun

**Affiliations:** College of Energy, Soochow Institute for Energy and Materials InnovationS (SIEMIS), Key Laboratory of Advanced Carbon Materials and Wearable Energy Technologies of Jiangsu Province, Soochow University, 215006 Suzhou, Jiangsu People’s Republic of China

**Keywords:** 3D printing, NiCoP/MXene, Asymmetric supercapacitor, Energy density, Tailorable loading

## Abstract

Designing high-performance electrodes via 3D printing for advanced energy storage is appealing but remains challenging. In normal cases, light-weight carbonaceous materials harnessing excellent electrical conductivity have served as electrode candidates. However, they struggle with undermined areal and volumetric energy density of supercapacitor devices, thereby greatly impeding the practical applications. Herein, we demonstrate the in situ coupling of NiCoP bimetallic phosphide and Ti_3_C_2_ MXene to build up heavy NCPM electrodes affording tunable mass loading throughout 3D printing technology. The resolution of prints reaches 50 μm and the thickness of device electrodes is ca. 4 mm. Thus-printed electrode possessing robust open framework synergizes favorable capacitance of NiCoP and excellent conductivity of MXene, readily achieving a high areal and volumetric capacitance of 20 F cm^−2^ and 137 F cm^−3^ even at a high mass loading of ~ 46.3 mg cm^−2^. Accordingly, an asymmetric supercapacitor full cell assembled with 3D-printed NCPM as a positive electrode and 3D-printed activated carbon as a negative electrode harvests remarkable areal and volumetric energy density of 0.89 mWh cm^−2^ and 2.2 mWh cm^−3^, outperforming the most of state-of-the-art carbon-based supercapacitors. The present work is anticipated to offer a viable solution toward the customized construction of multifunctional architectures via 3D printing for high-energy-density energy storage systems.

**Electronic supplementary material:**

The online version of this article (10.1007/s40820-020-00483-5) contains supplementary material, which is available to authorized users.

## Highlights

- Utilizing 3D printing allows the fine construction of electrodes with tailorable thickness and precise tuning of mass loading of active materials.
- 3D-printed NiCoP/MXene//AC asymmetrical supercapacitor full cells harvest a record-high areal/volumetric energy density of 0.89 mWh cm^−2^/2.2 mWh cm^−3^.

**Electronic supplementary material:**

The online version of this article (10.1007/s40820-020-00483-5) contains supplementary material, which is available to authorized users.

## Graphical abstract

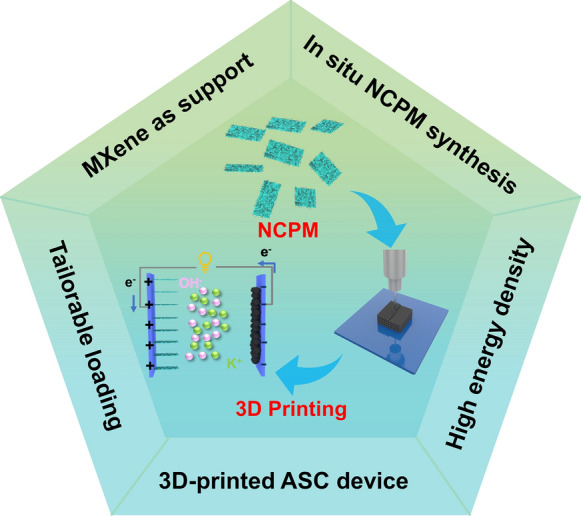

## Introduction

Utilizing advanced manufacturing technology to achieve tailorable electrode configurations plays a key role in boosting the electrochemical performances of energy storage devices. Along this line, extrusion-based 3D printing, a cost-effective and versatile technique relying on a three-axis motion stage to create well-defined periodic geometries via layer-by-layer stacking, has readily been employed in energy storage realm [1–5]. In contrast to traditional fabrication methods such as doctor blade coating, the thickness of electrodes and loading of active materials can be in target adjusted by the ink property, printing speed, and/or number of printed layers, thereby enhancing both energy density and power density of thus-constructed devices [6–8].

Recent years have witnessed an astounding increase in academic studies pertaining to 3D-printed supercapacitors, owing to their high power density, long lifespan, and excellent reliability [9, 10]. In this respect, carbonaceous materials manifest high electrical conductivity, ideal rheological property, structural diversity, and low cost, which are in favor of formulating printable inks to customize electrodes for supercapacitors [5, 11–13]. Of note, the porous structure of as-printed architecture would facilitate the electrolyte infiltration and ion transport, and ultimately benefit for the improvement of device performances [14, 15]. For instance, a 3D-printed symmetric supercapacitor based on activated carbon/carbon nanotube/reduced graphene oxide (AC/CNT/rGO) electrode managed to deliver areal and volumetric energy density of 0.63 mWh cm^−2^ and 1.43 mWh cm^−3^, respectively [12]. In further contexts, according to the equation *E* = 1/2 *CV*^2^, the energy density (*E*) of a supercapacitor is mediated by active material capacitance (*C*) and device operation voltage (*V*). On one hand, the capacitance value is mainly governed by the intrinsic properties of electrode materials. On the other hand, the voltage can be maximized by matching two electrodes with distinct voltage windows in an asymmetric cell configuration, further augmenting the energy density [16, 17]. Pioneer studies have demonstrated that asymmetric supercapacitor (ASC) devices can be constructed by 3D printing of rGO-supported hybrid inks (e.g., VO_*x*_/rGO//VN/rGO [18], G/ZnV_2_O_6_@Co_3_V_2_O_8_//G/VN [19]), readily harvesting favorable gravimetric and areal energy densities. Nevertheless, the lightweight feature of carbon-based electrodes greatly compromises the volumetric energy density of energy storage systems toward practical applications. Consequently, further efforts need to be devoted to searching for weighted and conductive support to replace rGO. MXene (Ti_3_C_2_T_*x*_) harnessing high tap density (~ 4 g cm^−3^) and good electrical conductivity (~ 6500 S cm^−1^) has emerged as a desirable candidate [20, 21]. However, 3D printing of MXene-supported composites to customize ASC full cells targeting high volumetric energy density has not been achieved thus far.

In this contribution, we report a well-designed ASC device affording high volumetric energy density that is realized throughout 3D printing. Such an ASC cell encompasses a printed NiCoP/MXene (NCPM) positive electrode and a printed AC negative electrode, wherein the NCPM architecture is created via in situ growth of NiCoP nanowires on the surface of Ti_3_C_2_T_*x*_ nanosheets. Utilizing 3D printing would allow the fine construction of electrodes with tailorable thickness and precise tuning of mass loading of active materials. In turn, the 3D-printed ASC enables a fast charge–discharge rate, a wide voltage window of 1.4 V, and a long cycle life up to 5000 cycles. More significantly, it readily harvests an ultrahigh areal and volumetric energy density of 0.89 mWh cm^−2^ and 2.2 mWh cm^−3^, respectively. Our work demonstrates that advanced printing technology holds great promise in customizing high volumetric energy density electrodes for next-generation practical energy storage systems.

## Experimental Section

### Synthesis of Ti_3_C_2_T_*x*_ (MXene) Nanosheets

Ti_3_AlC_2_ precursor was purchased from JiLin 11 Technology Co., Ltd. To prepare MXene nanosheets, 2 g of LiF was added to 40 mL of HCl (9 M) and stirring about 30 min, followed by the slow addition of 2 g of Ti_3_AlC_2_. After etching at 35 °C for 24 h, the obtained product was washed using deionized water for several times until the pH of the solution is around 6. After 2 h sonication under Ar flow and 1 h centrifugation at 3500 rpm to separate the sediment, an aqueous dispersion containing the MXene nanosheets (2 mg mL^−1^) was finally obtained.

### Preparation of NiCoP and MXene (NCPM) Composite

In a typical preparation, 0.7 g CoCl_2_·6H_2_O, 0.35 g NiCl_2_·6H_2_O (Co/Ni molar ratio 2:1), and 0.5 g urea were dissolved in 30 mL deionized water under vigorous stirring until completely dissolved. Next, prepared MXene dispersions (30 mL; 2 mg mL^−1^) were added into the solution in a drop-by-drop manner and stirred for 30 min under Ar flow. Thus-formed homogeneous dispersion was then transferred into a 100-mL Teflon-lined stainless-steel autoclave and kept at 120 °C for 10 h. Upon centrifugation and freeze-drying, the product was collected for further phosphorization to attain NCPM: 0.15 g NiCo/MXene-precursor and 1 g NaH_2_PO_2_ were placed into two individual ceramic boats and then positioned in a quartz tube (NaH_2_PO_2_ at the upstream side). The quartz tube was subsequently heated to 300 °C under a N_2_ atmosphere by a tube furnace with a temperature ramping speed of 2 °C min^−1^. Experiencing thermal annealing at 300 °C for 2 h, NCPM was finally obtained.

### Ink Formulation for 3D Printing

CNT suspensions (8 wt%) were purchased from QingDao HaoXin New Energy Technology Co. LTD. A high concentrated CNT dispersion (18 wt%) can be attained via a simple evaporation process. To prepare the ink with suitable viscosity for 3D printing, 1.67 g CNT suspension (18 wt%) and 0.7 g NCPM were mixed together (with an actual mass ratio of 3:7) without adding any polymer binder to form a high viscosity slurry (NCPM/CNT ink). Similarly, AC/CNT ink can be produced by employing 1.67 g CNT suspension (18 wt%) and 0.7 g AC powder (with an actual mass ratio of 3:7).

### Electrode Assembly via 3D Printing

As-formulated ink was loaded into a 3-mL syringe barrel and centrifuged for 3 min at 2500 rpm to remove the inside air bubbles prior to printing. In detail, the 3-mL syringe barrel was positioned into a 50-mL centrifuge tube for carrying out centrifugation. The ink was extruded through a needle with an inner diameter of 210 μm by air pressure, which was controlled by a benchtop robot (QZ-NC0903). The optimal extrusion pressure was 20–35 psi. The movement speed of the nozzle was 2–4 mm s^−1^. Various designed patterns based on NCPM ink and AC ink can be directly printed on polyethylene terephthalate (PET) substrate with a preset line spacing of 200–400 μm and a maintained nozzle height of ca. 200 μm. The height of the electrodes was varied from 0.3 to 2 mm.

### Characterizations

Scanning electron microscopy (SEM) images were acquired using a Hitachi SU8010 scanning electron microscopy. The detailed structure and corresponding elemental compositions were examined by transmission electron microscopy (TEM) (Titan Themis Cubed G2 300; Tecnai G2 F20 S-TWIN 27). X-ray diffraction (XRD) patterns were collected using a Bruker D8 Advance Diffractometer. X-ray photoelectron spectroscopy (Escalab 250Xi) was employed to analyze the surface chemistry of samples.

### Electrochemical Measurements

All electrochemical measurements including cyclic voltammetry (CV) and electrochemical impedance spectroscopy (EIS) were carried out using a CHI 660 electrochemical workstation. As for three-electrode configuration measurement, 3D printed electrode, graphite foil, and saturated calomel electrode (SCE) were used as a working electrode, counter electrode, and reference electrode, respectively. As for two-electrode configuration (ASC), printed NCPM and printed AC electrode were served as the positive electrode and negative electrode, respectively. Gravimetric capacitance (*C*_G_), areal capacitance (*C*_A_), and volumetric capacitance (*C*_V_) of the single printed electrode and the printed ASC devices can be calculated based on the galvanostatic charge/discharge (GCD) curves using Eqs. (1), (2), and (3), respectively.

1 $$C_{\text{G}} = \frac{I\Delta T}{m\Delta U}$$

2 $$C_{\text{A}} = \frac{I\Delta T}{A\Delta U}$$

3 $$C_{\text{V}} = \frac{I\Delta t}{V\Delta U}$$

where *C*_G_, *C*_A_, and *C*_V_ are the gravimetric, areal and volumetric capacitance (F g^−1^, F cm^−2^, and F cm^−3^), Δ*U* is the potential window (*V*), *I* is the discharge current (*A*), Δ*t* is the discharge time (*s*), m is the mass loading of the materials on the electrode (*g*), *A* is the geometric working area of the electrode (cm^2^), and *V* is the volume of the electrode (cm^3^). For the ASC devices, the areal and volumetric energy density (*E*, mWh cm^−2^ and mWh cm^−3^) can be, respectively, calculated by Eqs. (4) and (5):

4 $$E_{A} = \frac{1000}{2 \times 3600}C_{\text{A}} \left( {\Delta U} \right)^{2}$$

5 $$E_{V} = \frac{1000}{2 \times 3600}C_{\text{V}} \left( {\Delta U} \right)^{2}$$

where *C*_A_ is the areal capacitance (F cm^−2^), *C*_V_ is the volumetric capacitance (F cm^−3^), and *U* is the working voltage (*V*).

## Results and Discussion

### Preparation and Characterization of NCPM

**Fig. 1:**
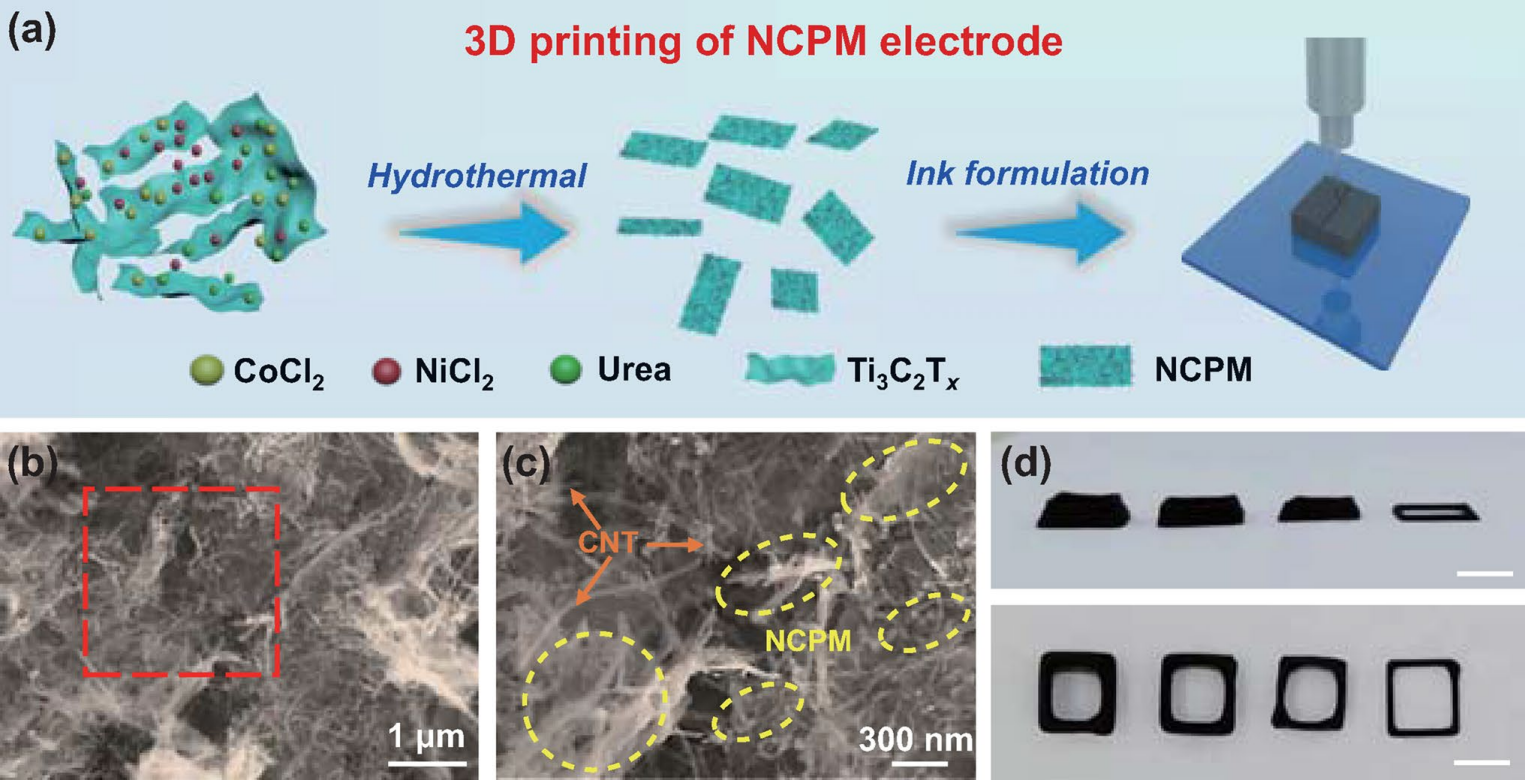
**a** Schematic illustration showing the NCPM synthesis and the 3D printing process. **b**, **c** SEM images of the NCPM composite ink. **d** Digital photos showing printed hollow square lattices with increasing wall thickness. Scale bar: 8 mm

Figure 1a illustrates the synthesis of NCPM composites and the 3D printing process. Few-layered Ti_3_C_2_T_*x*_ flakes were firstly prepared by using Ti_3_AlC_2_ precursor via a wet etching procedure (HCl + LiF) and subsequent ultrasonication in Ar atmosphere for 2 h (Fig. S1). Note that the as-formed Ti_3_C_2_T_*x*_ flakes are negatively charged with terminal groups (–F, –OH, –O, etc.), which could serve as the perfect support for attracting positively charged metal ions (Ni^2+^, Co^2+^) [22, 23]. Upon the addition of Ti_3_C_2_T_*x*_ flakes into aqueous dispersion containing urea, NiCl_2_, and CoCl_2_, co-precipitation of NiCo-layered double hydroxides (LDH) on the surface of Ti_3_C_2_T_*x*_ nanosheets occurred throughout hydrothermal treatment. Further thermal annealing with the presence of NaH_2_PO_2_ at 300 °C would ultimately give rise to the formation of NCPM composites. Accordingly, the NCPM-based ink for 3D printing can be fabricated by mixing thus-produced NCPM with CNT dispersion at an optimized mass ratio (details presented in the Experimental Section). It can be observed from the high magnification SEM images that both NCPM and CNT co-exist in the ink without discernible aggregation (Fig. 1b, c). The CNT in the ink acts as a binder and conductive agent accompanied by porous architecture, which could promote electrolyte penetration and ion diffusion. Thus-prepared ink can be extruded through a micro-nozzle to pattern different geometries on certain substrates. Figure S2 presents the digital photos of various patterns and shapes realized by 3D printing of such NCPM ink. Moreover, hollow square lattices with increasing wall thickness can also be printed (Fig. 1d), demonstrating the versatility of 3D printing in building-up multiple patterns with high accuracy and productivity.

**Fig. 2:**
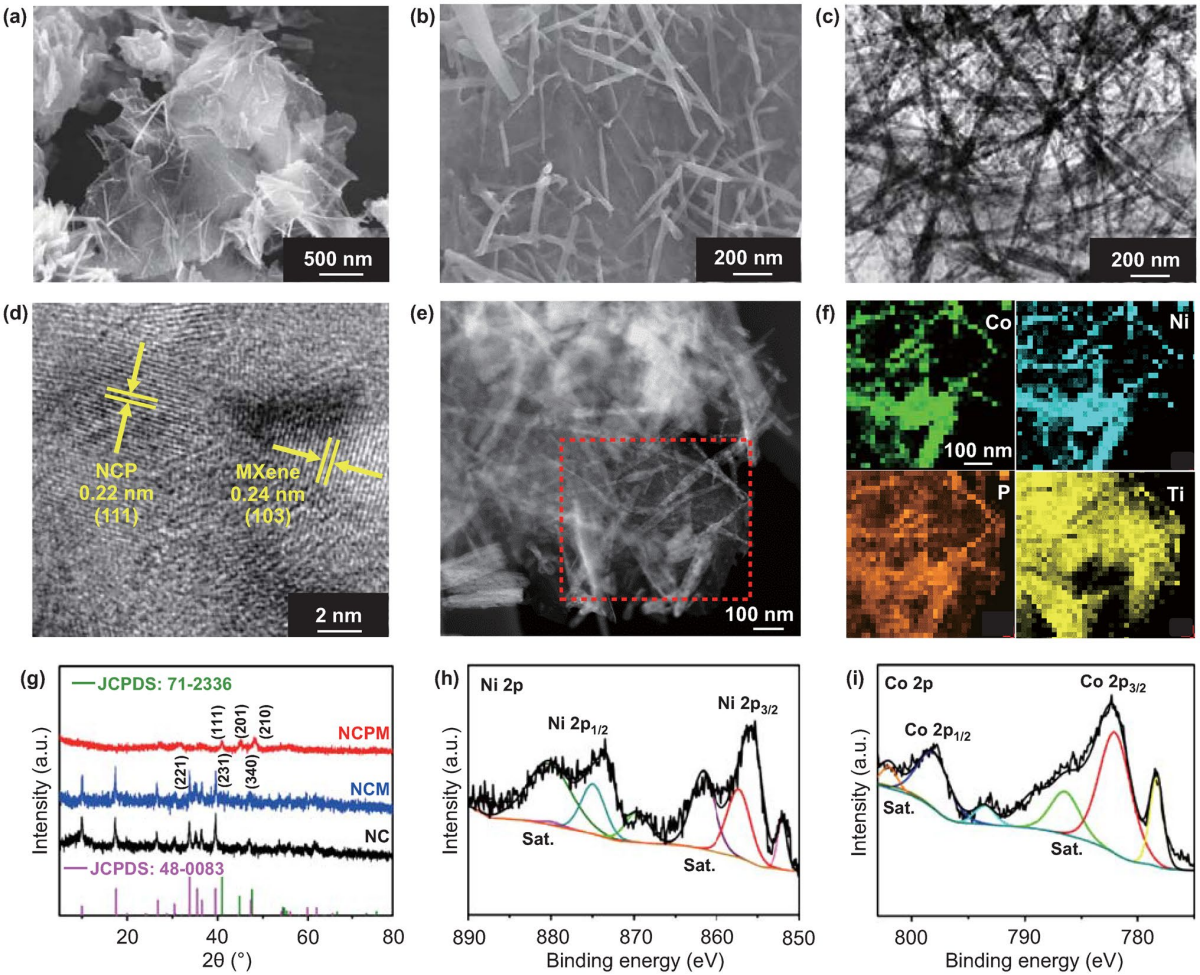
**a**, **b** SEM images of NCPM composites. **c** Low-magnification TEM and **d** HRTEM images of NCPM corroborating the tight assembly of MXene and NiCoP. **e** STEM image and **f** elemental maps of NCPM. **g** XRD patterns of NC, NCM, and NCPM. **h** Ni 2p and **i** Co 2p XPS spectra of NCPM

The detailed microstructures of prepared NCPM composites were probed by SEM and TEM. SEM observations in Fig. 2a, b manifest that wire-shaped NiCoP with an average width of 40 nm is distributed on Ti_3_C_2_T_*x*_ nanosheets. It is interesting to note that the synthesis of NiCo-LDH species in the absence of MXene supports would result in sphere-like morphologies (Fig. S3); in other words, MXene flakes possessing numerous surface functional groups could promote the nucleation and growth of tubular structures to form NiCo-LDH/MXene (NCM) [24, 25]. Meanwhile, the in situ phosphorization treatment retains the morphology of NCPM similar to that of NCM (Fig. S4). Low-magnification TEM inspections further exhibit the tight assembly of Ti_3_C_2_T_*x*_ flakes and NiCoP nanowires (Figs. 2c and S5), agreeing well with the SEM results. The high-resolution TEM (HRTEM) image (Fig. 2d) discloses a well-defined lattice fringe with a distance of 0.24 nm and 0.22 nm, corresponding to the (103) plane of MXene and (111) plane of NCP, respectively [23, 26]. In addition, scanning transmission electron microscopy (STEM) imaging and elemental mapping were carried out, readily disclosing homogeneous distributions of Ni, Co, P, and Ti elements within a probed area (Fig. 2e, f).

To further examine the crystal structures and chemical compositions of as-synthesized NCPM, XRD and X-ray photoelectron spectroscopy (XPS) analysis were carried out. Figure 2g displays the XRD patterns of NC, NCM, and NCPM, where the diffraction peaks accord well with NiCo-LDH (JCPDS No. 48-0083) and NiCoP (JCPDS No. 71-2336), respectively [17, 27]. Meanwhile, the typical signal of (002) plane of exfoliated MXene shifts down to 6.7° (from etched MXene at 9.1°), indicative of augmented interlayer spacing (Fig. S6), which is in good agreement with previous reports [28, 29]. Atomic force microscopy (AFM) analysis further confirms the ultrathin nature of the exfoliated MXene nanosheets, displaying a survey thickness of 3.5 nm (Fig. S7). XPS survey spectrum suggests the presence of Ni, Co, and P elements after the NiCoP growth (Fig. S8). As for the high-resolution Ni 2p spectrum in Fig. 2h, the peaks locating at 852.3/870.0, 857.7/874.9, and 861.7/880.8 eV can be attributed to the Ni–P, oxidized Ni species, and satellite signals, respectively [30]. Figure 2i shows the Co 2p XPS spectrum, where Co–P bonding, oxidized Co, and satellite peaks can be found [17]. Moreover, the high-resolution Ti 2p spectrum can be deconvoluted into four peaks at the binding energy of 464.2, 461.5, 458.8, and 455.4 eV, corresponding to Ti–O, Ti–O_*x*_, Ti–X, and Ti–C, respectively (Fig. S9a). Meanwhile, with respect to the P 2p spectrum, the peaks at 128.8 and 129.9 eV are ascribed to the P 2p_1/2_ and P 2p_3/2_ signals in NiCoP (Fig. S9b). The broad peak at 133.8 eV is indicative of oxidized phosphorus species, which might be due to the interconnection with Ti_3_C_2_ [23]. The XPS data demonstrate the successful integration between Ti_3_C_2_ and NiCoP.

## 3D Printing Process

**Fig. 3:**
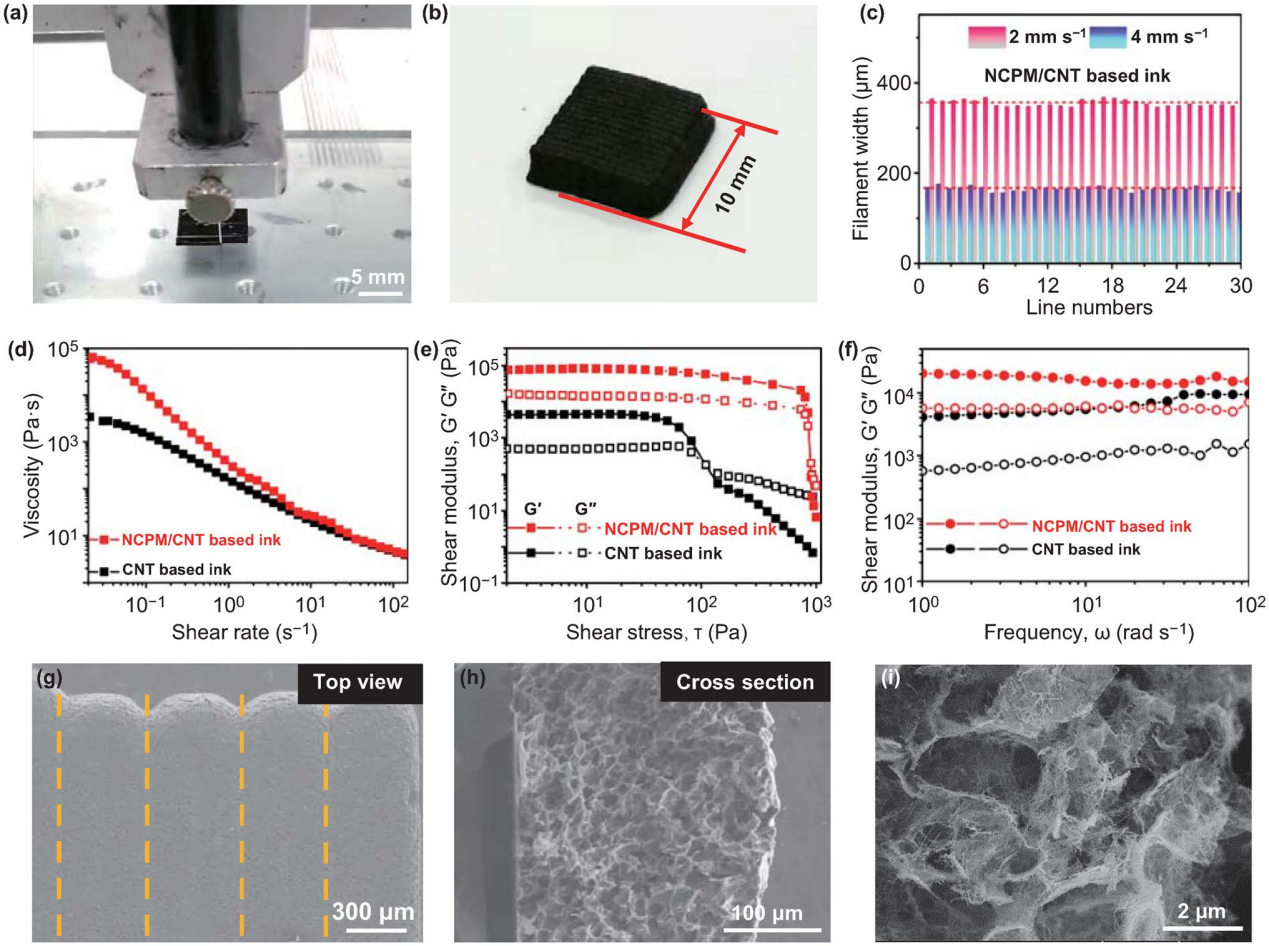
**a** Photograph of the 3D printing setup. **b** Photograph showing the 3D-printed square lattice. **c** Width distributions of printed filaments by adopting two different printing speeds. **d** Apparent viscosity of as-fabricated NCPM/CNT and CNT inks as a function of shear rate. Storage modulus (G′) and loss modulus (G″) of NCPM/CNT and CNT inks as a function of **e** shear stress and **f** angle frequency. **g** Top-view and **h** cross-section SEM images of the printed NCPM electrode. **i** SEM image displaying the microstructure of the NCPM electrode after freeze-drying

As shown in Fig. 3a, 3D printing can be enabled by employing a benchtop robot with a three-axis stage controlled via programming. Accordingly, a square-shaped box with a dimension of 10 × 10 × 3 mm^3^ can be constructed in a layer-by-layer printing manner (Fig. 3b), where NCPM-based ink was injected from a nozzle at a constant speed of 2 mm s^−1^. Figure 3c manifests the printing capability and stability of our 3D printing process. As such, the width of printing filaments can be easily controlled by the printing speed, resulting in the average line at ca. 355 μm at a speed of 2 mm s^−1^. When the print speed was adjusted to 4 mm s^−1^, the average width falls down to ca. 165 μm. For both cases, there are no obvious fluctuations in the width of as-printed filaments. This can be further evidenced by the optical microscopy (OM) observation (Fig. S10). Note further that the line width of the electrode is normally measured after the evaporation process. Upon maintaining the identical printing pressure and nozzle size, a lower printing speed means that more ink can be deposited on the substrate (Fig. S11a). When the filament width is altered, the surface smoothness otherwise shows no significant change (Fig. S11b). In addition, when the printing pressure is kept identical, the formation of a dense microstructure is not related to the printing speed, as witnessed by the SEM inspection (Fig. S11c–f). Herein, the mass loading of printed architectures (e.g., electrodes) is dictated by the number of printed layers. To successfully build-up 3D-printed architectures with structural robustness, the rheological properties of as-prepared inks were evaluated. As shown in Fig. 3d, both inks (CNT based and NCPM/CNT based) exhibit a shear-thinning non-Newtonian fluid behavior. The viscosity declines upon the increase of shear rates, which enables the inks to flow continuously throughout the nozzle. It can be observed that the viscosity tends to infinity at a very low shear rate (below 0.1 s^−1^), implying that the inks behave as a Bingham plastic with yield stress [3]. Such a feature can be attributed to the addition of CNT suspension. In contrast, the ink without adding CNT would behave like a flowing liquid (Fig. S12). Moreover, the storage modulus (G′) and loss modulus (G″) of both inks were examined as a function of stress amplitude (Fig. 3e). Obviously, the yield stress value of the NCPM/CNT-based and CNT-based ink lies in ca. 800 and 110 Pa, respectively. Below the crossover point (G′ > G″), both inks present dominant elastic-like solid behavior. With the increase of shear stress to result in G′ < G″, solid to liquid transition occurs, where the ink is favorable to be extruded from the nozzle [31, 32]. Figure 3f further shows the G′ and G″ of both inks as a function of frequency. In the range of frequency sweep, both inks display higher G′ as compared to G″; in addition, G′ and G″ afford a frequency-independent feature [18]. This indicates that both inks are of high dispersion stability and can be stored for a long term. Note that the AC/CNT-based ink could demonstrate the similar rheological properties (Fig. S13) that are desirable for 3D printing.

The detailed morphology of 3D-printed NCPM/CNT electrodes at a microscopic scale was inspected by SEM. Figure 3g shows the top view, low-magnification SEM image, revealing the electrode possessing one-layer thickness is constituted by continuous and tightly stacked filaments. A close-up cross-sectional view in Fig. 3h indicates that the layer thickness is ca. 260 μm. More interestingly, owing to experiencing the water removal by freeze-drying, thus-printed NCPM/CNT electrode is featured by interconnected structures with the presence of ample hierarchical pores (Fig. 3i). The specific surface area of the NCPM/CNT according to the Brunauer–Emmett–Teller method possesses a value of 102.9 m^2^ g^−1^ with an average pore size of 22.8 nm (Fig. S14). This would ultimately be beneficial to ion transport and electrolyte penetration [33].

### Electrochemical Performance of 3D-printed NCPM Electrode

**Fig. 4:**
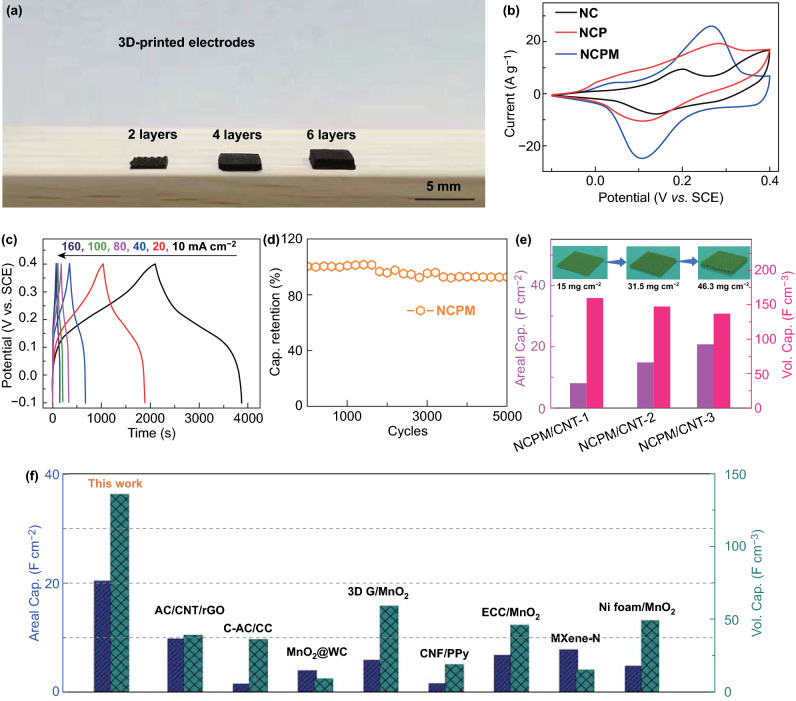
**a** Photograph showing the thickness of the 3D-printed NCPM electrodes. **b** CV profiles of NC, NCP, and NCPM tested in a three-electrode configuration. **c** GCD curves of printed NCPM/CNT electrode at different current densities. **d** Cycling performance of printed NCPM/CNT electrode. **e** Statistics of areal and volumetric capacitances for the printed NCPM/CNT electrodes possessing different layers. Inset: Schematic illustration of NCPM/CNT electrodes with different thicknesses and mass loading. **f** Comparison of the areal and volumetric capacitances between this work and other reported systems

With the aid of 3D printing technology, NCPM/CNT electrodes with different layer numbers can be constructed in a facile manner, as shown in the digital photo in Fig. 4a. In this regard, the mass loading and electrode thickness can be in turn adjusted. To demonstrate the advance in the electrochemical performance of NCPM, control samples including NiCo-LDH (NC) and NCP were evaluated. Figure 4b manifests the cyclic voltammetry (CV) profiles of NC, NCP, and NCPM in a three-electrode configuration at a scan rate of 10 mV s^−1^, where larger curve area indicates higher capacitance value. Based on the galvanostatic charge/discharge (GCD) curves at a current density of 1 A g^−1^ (Fig. S15), NCPM, NCP, and NC managed to deliver a specific capacitance of 1359, 920, and 608 F g^−1^, respectively. The advantage of NCPM over the NCP and NC lies in the rational incorporation of MXene scaffolds, which endows the hybrid electrodes with superb conductivity and sufficient open channels. In addition, the capacitance contribution from CNT in the electrode was also excluded to highlight the function of MXene via supplementary tests, as shown in Fig. S16a. The rate performances of both NCPM and NCP inks are further plotted (Fig. S16b), which are derived from the GCD curves at different current densities ranging from 1 to 10 A g^−1^. As such, NCPM displays superior rate capability (with a higher retention of 79.6%) to that of NCP (69.1%).

The electrochemical performances of 3D-printed NCPM electrodes affording different layer thicknesses at 0.5, 1.0, and 1.5 mm, denoted, respectively, as NCPM/CNT-1, NCPM/CNT-2, and NCPM/CNT-3, were accordingly investigated. Figure 4c presents the GCD curves of NCPM/CNT-1 measured between − 0.1 and 0.4 V at various current densities from 10 to 160 mA cm^−2^. Symmetric charge–discharge profiles can be clearly observed, implying facile charge transport kinetics and high Coulombic efficiencies of devices. Meanwhile, the NCPM/CNT electrode demonstrates favorable cyclic stability with a capacitance retention of over 90% after 5000 cycles at a current density of 100 mA cm^−2^ (Fig. 4d).

Figure 4e summarizes the derived areal/volumetric capacitance values of these printed electrodes (increased mass loadings of 15, 31.5, and 46.3 mg cm^−2^ corresponding to NCPM/CNT-1, NCPM/CNT-2, and NCPM/CNT-3 electrodes, respectively). A thicker electrode normally harnesses a higher mass loading, hence corresponding to a higher areal capacitance. Nevertheless, the volumetric capacitance might be affected to some extent for the relatively thick electrode because of the sluggish ion and electron transport. As such, the areal and volumetric capacitance of NCPM/CNT-3 could reach 20 F cm^−2^ and 137 F cm^−3^, respectively. These values are markedly superior to those from the state-of-the-art systems, including AC/CNT/rGO [12], commercial carbon black/carbon cloth (C-AC/CC) [12], MnO_2_@WC [34], 3D G/MnO_2_ [33], carbon nanofiber/polypyrrole (CNF/PPy) [35], exfoliated carbon cloth/MnO_2_ (ECC/MnO_2_) [36], MXene-N [29], and Ni foam/MnO_2_ [37] (Fig. 4f).

### Electrochemical Performance of 3D-printed NCPM//AC ASC Device

**Fig. 5:**
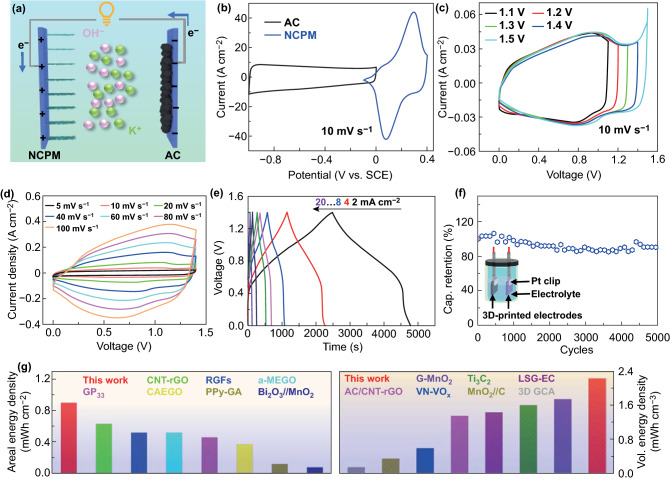
**a** Schematic illustration of the constructed ASC full cell based on 3D-printed NCPM positive electrode and 3D-printed AC negative electrode. CV curves of **b** printed NCPM and AC at a scan rate of 10 mV s^−1^. **c** NCPM//AC ASC full cells in different voltage windows. **d** NCPM//AC ASC full cell recorded at various scan rates. **e** GCD curves in the voltage range of 0–1.4 V at various current densities. **f** Cycling performance of the ASC full cell, with the inset illustrating the 3D-printed ASC tested in aqueous electrolyte. **g** Comparison of areal and volumetric energy density between our 3D-printed NCPM//AC ASC and other state-of-the-art symmetric/asymmetric capacitors

Since the positive and negative electrode for ASC was successfully developed via printing, the electrochemical performance of 3D-printed ASC devices was further explored. Figure 5a displays the schematic illustration of the device, comprising a printed NCPM positive electrode and a printed AC negative electrode in a KOH aqueous electrolyte. Figure 5b shows the individual potential windows, where the CV profiles of AC (− 1.0–0.0 V) and NCPM (− 0.1 to 0.4 V) electrodes were collected in 2 M KOH at the same scan rate of 10 mV s^−1^. To attain an optimized performance for ASC, the charge balance between the cathode and the anode should be satisfied with a calculated mass loading ratio of positive:negative electrode at 5:1 (Supporting Text). As shown in Fig. 5c, a collection of CV scans with an increasing potential window was carried out to probe the stable operation region of our ASC device. Obviously, a maximized stable window of 0–1.4 V was realized due to the effective suppression of oxygen evolution from the electrolyte, and hence was selected as the potential window of the full cell. Figure 5d manifests the CV curves of the assembled ASC cell recorded at various scan rates (from 5 to 100 mV s^−1^) with a working voltage from 0 to 1.4 V. Clearly, quasi-rectangular-shaped CV profiles couple with two board redox peak humps can be maintained, highly suggestive of the combination of double-layer capacitive and pseudocapacitive behavior. The GCD curves in the voltage range of 0–1.4 V in Fig. 5e show good symmetry at various current densities, demonstrating high Coulombic efficiency. The non-linear curves are the typical feature of a hybrid supercapacitor, agreeing well with CV results. On a basis of these tests, the specific capacitance of the printed ASCs was derived to be 2.76–3.29 F cm^−2^ (areal) and 9.2–10.97 F cm^−3^ (volumetric) at different current densities (Fig. S17). Figure 5f discloses the cycling performance at a current density of 12 mA cm^−2^. The full cell retains 87.5% of its initial specific capacitance value after 5000 GCD cycles, revealing superb cycling stability. Post-mortem SEM observations of NCPM electrodes upon 5000 cycles indicate the well preservation of hollow interiors (Fig. S18), implying good structural stability. More importantly, the printed electrodes manifest improved rate capability and cycling stability in comparison with bulk electrodes. As depicted in Fig. S19, the bulk and printed electrode could, respectively, retain 68.1% and 82.3% of its initial capacitance at 5 mV s^−1^. EIS measurements before and after 5000 cycles were carried out, revealing favorable electrochemical stability of printed ASC (Fig. S20). In the high-frequency region, Nyquist plots before and after cycling reflect good conductivities of the electrode. The equivalent circuit model indicates that the charge transfer resistance (*R*_ct_) augments to a reasonable value after 5000 cycles. In the low-frequency region, the slopes for the lines show a slight change upon cycling, implying stable ion diffusivity in the electrode. Accordingly, our 3D-printed NCPM//AC asymmetric supercapacitors with tunable electrode thickness enable a maximum areal energy density of 0.89 mWh cm^−2^, outperforming many emerging energy storage devices encompassing CNT–rGO [12], RGF [38], activated microwave-expanded graphite oxide (a-MEGO) [39], graphene/PEDOT:PSS (GP_33_) [40], chemical-activated exfoliated graphite oxide (CAEGO) [41], polypyrrole-coated graphene aerogel (PPy-GA) [42], and Bi_2_O_3_//MnO_2_ [43]. More significantly, the volumetric energy density of the 3D-printed full cells can reach 2.2 mWh cm^−3^, which is evidently superior to those of G-MnO_2_ [33], Ti_3_C_2_ [44], laser-scribed graphene (LSG) [45], CNT–rGO [12], VN–VOx [18], MnO_2_/C [46], and 3D graphene composite aerogel (3D GCA) [47]. In detail, a comparison of volumetric energy density realized between this work and recently reported supercapacitor systems are drawn in Table S1.

## Conclusions

In summary, we have demonstrated the 3D-printed design of NiCoP/MXene//AC asymmetrical supercapacitor full cells to harvest a record-high volumetric energy density. Distinct from the employment of universal light-weight carbon structures as electrode materials, utilizing heavy NiCoP/MXene hybrids serves as one of the feasible technological solutions to construct compact electrodes with high tap density. Meanwhile, a 3D-printed NCPM electrode with hierarchical pores and tunable mass loadings possesses facile charge transport and thorough electrolyte penetration, endowing it with improved rate capability and cycling stability. As a result, assembled ASC devices manage to deliver a high areal and volumetric energy density of 0.89 mWh cm^−2^ and 2.2 mWh cm^−3^, respectively. Such superb electrochemical performances corroborate the advance of 3D-printed, carbon-free NCPM materials as supercapacitor electrodes to realize high volumetric energy density. Our work offers a general strategy for the 3D-printed design of versatile electrode architectures targeting high-energy-density energy storage systems.

## Electronic Supplementary Material

Below is the link to the electronic supplementary material.

Supplementary material 1 (PDF 1093 kb)
