# Supplementary material for: 3D Printing of NiCoP/Ti3C2 MXene Architectures for Energy Storage Devices with High Areal and Volumetric Energy Density
Source: Nanomicro Lett. 2020 Jul 9;12:143. doi: 10.1007/s40820-020-00483-5 (PMC7770976; doi:10.1007/s40820-020-00483-5)
Supplement: Supplementary file 1 — Supplementary material 1 (PDF 1093 kb) [file 40820_2020_483_MOESM1_ESM.pdf]

Supporting Information for

3D Printing of NiCoP/Ti_3C_2 MXene Architectures for Energy Storage Devices with High Areal and Volumetric Energy Density

Lianghao Yu[1], Weiping Li[1], Chaohui Wei[1], Qifeng Yang[1], Yuanlong Shao[1], Jingyu Sun[1, *]

[1]College of Energy, Soochow Institute for Energy and Materials InnovationS (SIEMIS), Key Laboratory of Advanced Carbon Materials and Wearable Energy Technologies of Jiangsu Province, Soochow University, Suzhou, Jiangsu 215006, People's Republic of China

*Corresponding author. E-mail: sunjy86@suda.edu.cn (Jingyu Sun)

S1 Supplementary Figures

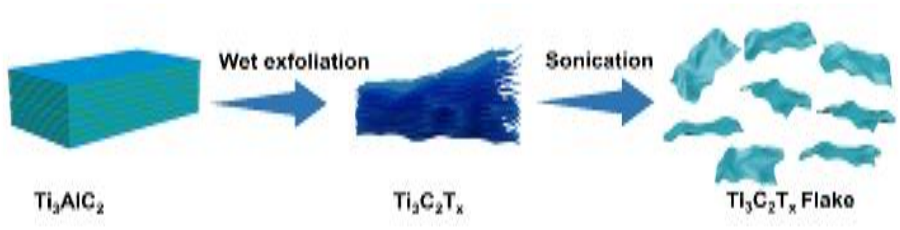

Fig. S1 Scheme showing the fabrication process of Ti_3C_2 MXene nanoflakes

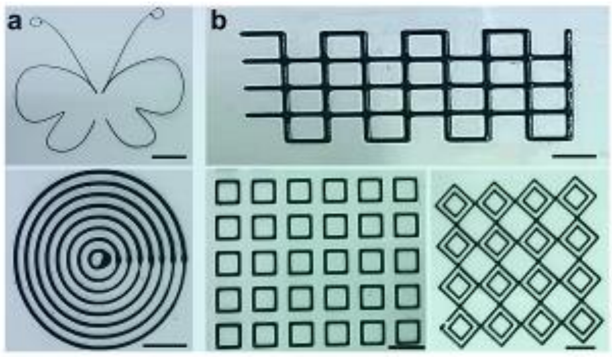

Fig. S2 (**a, b**) Digital photos showing various patterns and shapes realized by 3D printing. Scale bars: 5 mm

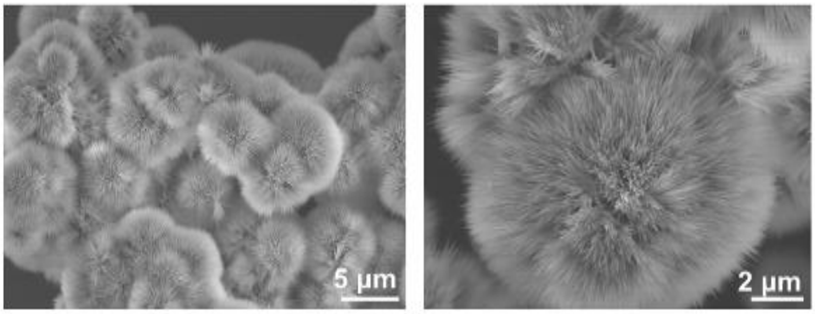

Fig. S3 SEM images showing the morphology (urchin-like aggregation) of bare NiCo-LDH in the absence of Ti_3C_2

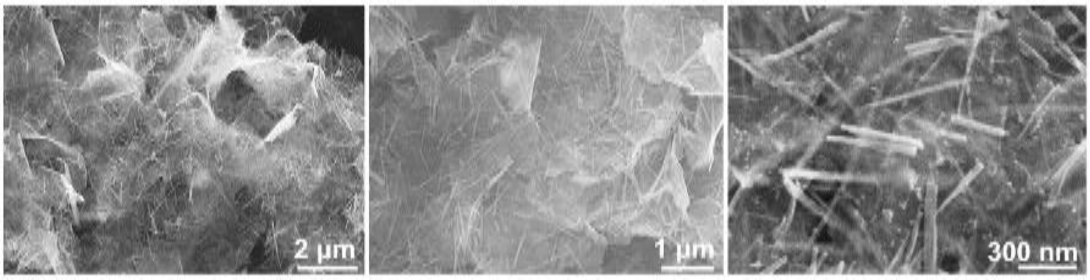

Fig. S4 SEM images showing the morphology of NiCo-LDH/Ti_3C_2 (NCM)

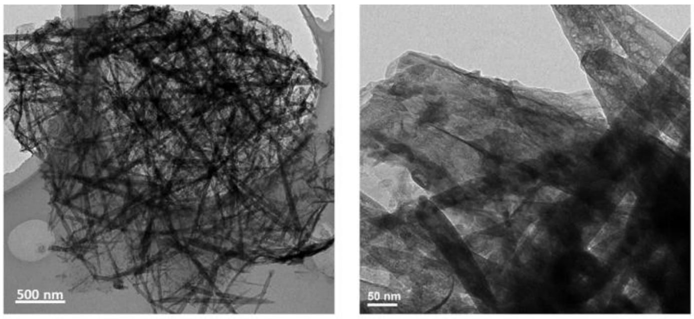

Fig. S5 Low-magnification TEM images of NiCoP/Ti_3C_2 (NCPM)

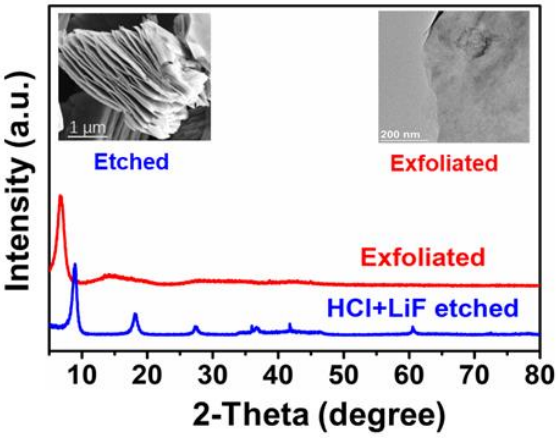

Fig. S6 XRD patterns of etched and exfoliated MXene. Inset showing the SEM image of etched sample (left) and TEM image of exfoliated MXene (right)

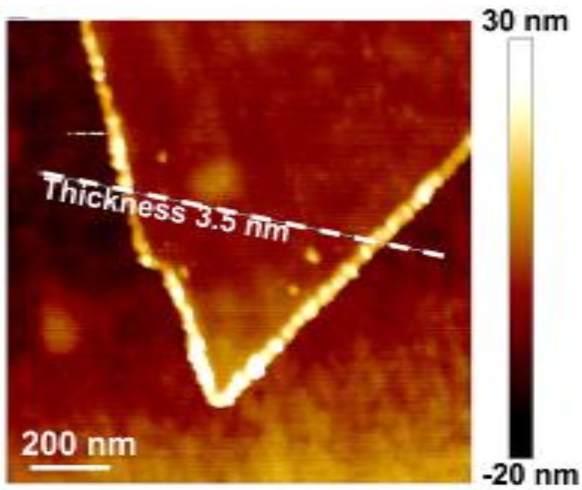

Fig. S7 Representative AFM image of exfoliated MXene flake

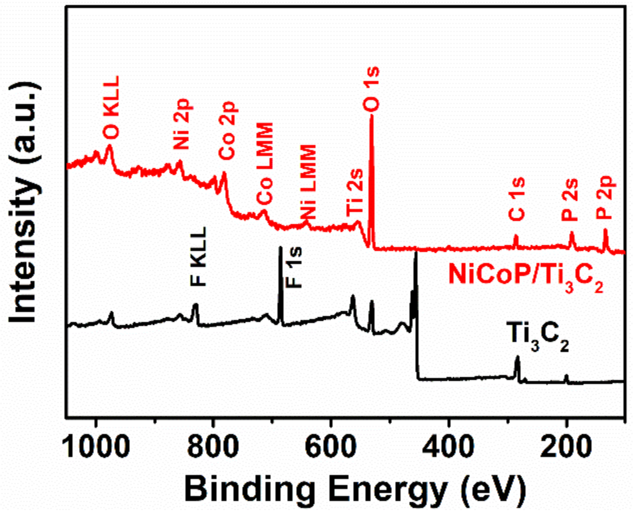

Fig. S8 XPS survey spectra of NCPM and Ti_3C_2 MXene

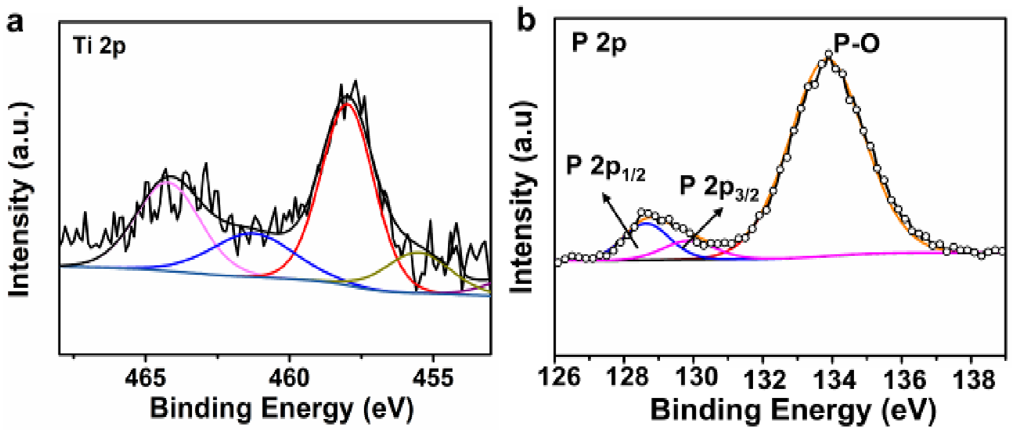

Fig. S9 (**a**) XPS Ti 2p spectrum of NCPM. (**b**) XPS P 2p spectrum of NCPM

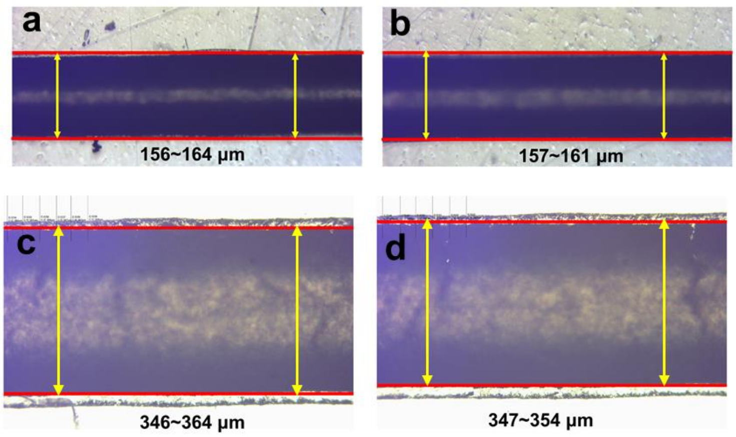

Fig. S10 Optical microscopy images of printed filaments at different printing speeds of (**a, b**) 4 mm s^{-1} and (**c, d**) 2 mm s^{-1}

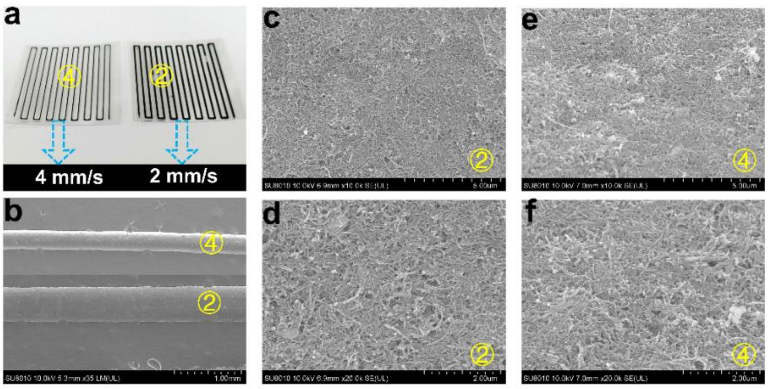

Fig. S11 (**a**) Real photo of the evaporation process (④ and ② denoting the printing speed at 4 and 2 mm s^{-1}, respectively). (**b**) Low magnification SEM image of filaments. (**c, d**) SEM images showing the microstructures of the ② filaments. (**e, f**) SEM images showing the microstructures of the ④ filaments

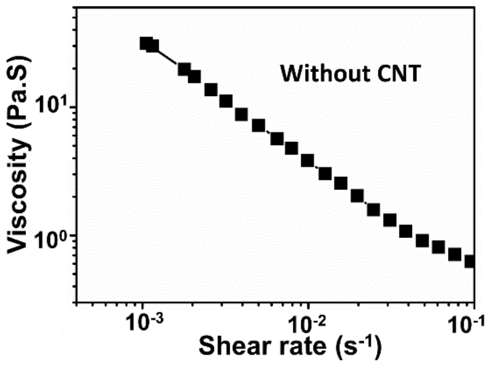

Fig. S12 Rheological property of the ink without adding CNT. Note that at a low shear rate (below 0.1 s^{-1}), the viscosity lies in the range of 0.6-30 Pa·s, which is not suitable for 3D printing

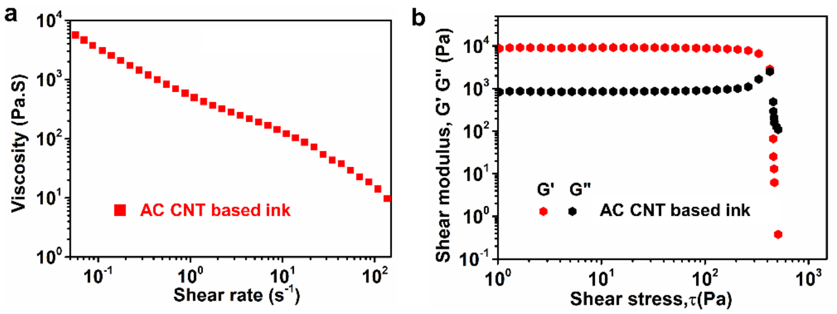

Fig. S13 Rheological properties of the AC/CNT ink

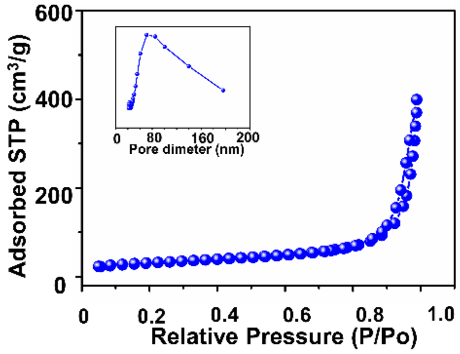

Fig. S14 N_2 adsorption/desorption isotherm and the corresponding pore size distribution (inset) of NCPM/CNT

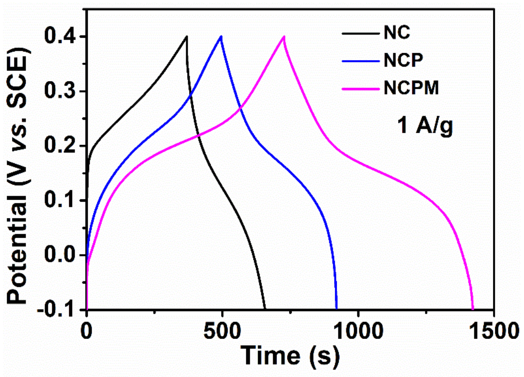

Fig. S15 GCD curves of the NCPM, NCP and NC at the current density of 1 A g^{-1}

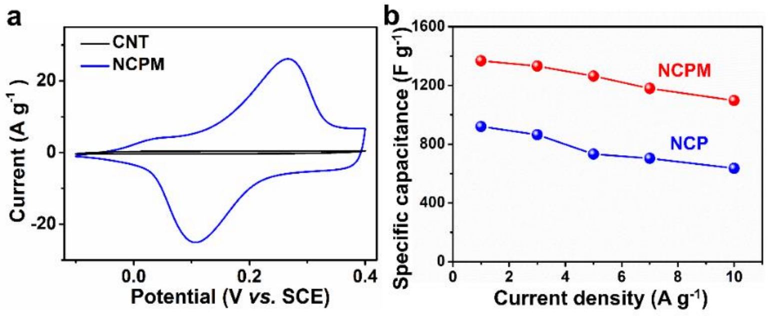

Fig. S16 (**a**) CV profiles of bare NCPM and bare CNT at a scan rate of 10 mV s^{-1}. (**b**) The measured capacitances of NCPM ink and NCP ink at different current densities

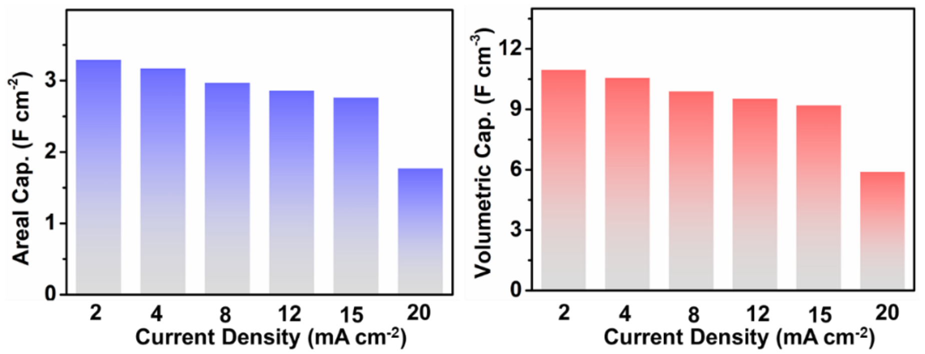

Fig. S17 Specific capacitances of the ASCs at different current densities

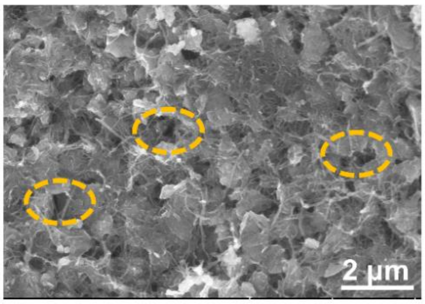

Fig. S18 SEM image of NCPM electrodes after 5000 cycles

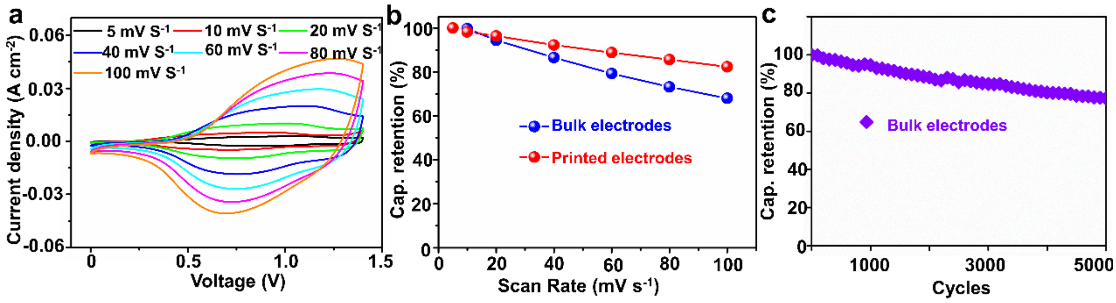

Fig. S19 (**a**) CV curves of the ASC full cell assembled using bulk electrodes at various scan rates. (**b**) Rate capability of the bulk electrodes and printed electrodes. (**c**) Cycling performance of the bulk electrodes

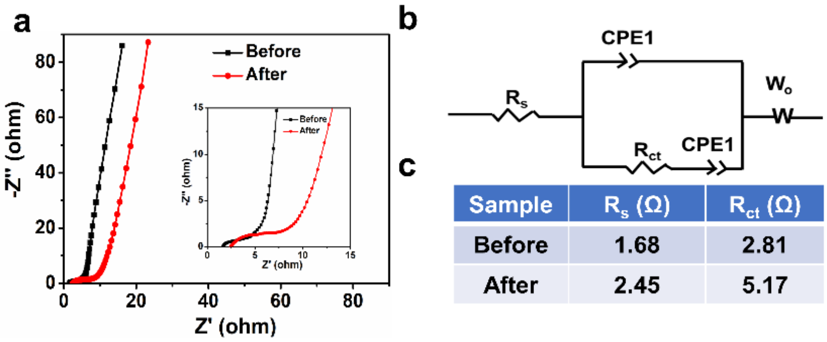

Sample	R_s (Ω)	R_{ct} (Ω)
Before	1.68	2.81
After	2.45	5.17

Fig. S20 EIS analysis of ASC device. (**a**) EIS profiles before and after 5000 cycles. (**b**) The Randles equivalent circuit model. (**c**) Fitting parameters of the ASC device

S2 Supplementary Text

The mass ratio between positive and negative electrodes can be determined by the following equation:

$$m^{+}/m^{-}=(C^{-}\times\Delta V^{-})/(C^{+}\times\Delta V^{+})$$

where m^{+}, C^{+}, and ΔV^{+} represent the mass, specific capacitance and potential window of the positive electrode material; m^{-}, C^{-}, and ΔV^{-} stand for the mass, specific capacitance and potential window of the negative electrode material. The optimized mass ratio of positive:negative electrode can be derived at ~5.

S3 Supplementary Table

Table S1 Comparison of volumetric energy density performances between this work and recently reported supercapacitor systems

Electrode material	$E_{volumetric}$ (mWh cm^{-3})	Asymmetric /Symmetric	Printing (Yes/No)	Refs.
**G-EC**	1.35	Symmetric	Yes	[S1]
**VN//VO$_x$**	0.60	Asymmetric	Yes	[S2]
**TiO_2/C//TiO_2/MnO_2**	0.30	Asymmetric	No	[S3]
**3D GCA**	0.15	Symmetric	Yes	[S4]
**VO$_x$/rGO//G-VN/rGO**	1.74	Asymmetric	Yes	[S5]
**C/CNT/rGO**	1.43	Symmetric	Yes	[S6]
**N-MCN/GH**	1.12	Symmetric	No	[S7]
**EGM**	1.40	Symmetric	No	[S8]
**(PEDOT)-cellulose paper**	1.00	Symmetric	No	[S9]
**TiN-Fe_2N**	0.61	Asymmetric	No	[S10]
**VN/CNT**	0.54	Symmetric	Yes	[S11]
**G/PANI-paper**	0.32	Symmetric	No	[S12]
**$Ni(OH)_2$**	0.59	Symmetric	Yes	[S13]
**B-LIG**	0.80	Symmetric	Yes	[S14]
**MWNT**	0.40	Symmetric	Yes	[S15]
**AC/CNT/MXene-N/rGO**	0.83	Symmetric	Yes	[S16]
**C/MnO_2**	0.20	Symmetric	No	[S17]
**δ-MnO_2**	0.18	Symmetric	Yes	[S18]
**$K_2Co_3(P_2O_7)_2$//Graphene**	0.96	Asymmetric	Yes	[S19]
**NCPM//AC**	2.2	Asymmetric	Yes	**This work**

Supplementary References

[1] M.F. El-Kady, V. Strong, S. Dubin, R.B. Kaner, Laser scribing of high-performance and flexible graphene-based electrochemical capacitors. Science **335**, 1326-1330 (2012). https://doi.org/10.1126/science.1216744

[2] K. Shen, J.W. Ding, S.B. Yang, 3D Printing quasi-solid-state asymmetric micro-supercapacitors with ultrahigh areal energy density. Adv. Energy Mater. **8**, 1800408 (2018). https://doi.org/10.1002/aenm.201800408

[3] X.H. Lu, M.H. Yu, G.M. Wang, T. Zhai, S.L. Xie, Y.C. Ling, Y.X. Tong, Y. Li, H-TiO_2@MnO_2//H-TiO_2@C core-shell nanowires for high performance and flexible asymmetric supercapacitors Adv. Mater. **25**, 267-272 (2013). https://doi.org/10.1002/adma.201203410

[4] C. Zhu, T.Y. Liu, F. Qian, T. Han, E.B. Duoss et al., Supercapacitors based on three-dimensional hierarchical graphene aerogels with periodic macropores. Nano Lett. **16**, 3448-3456 (2016). https://doi.org/10.1021/acs.nanolett.5b04965

[5] J.X. Zhao, Y. Zhang, X.X. Zhao, R.T. Wang, J.X. Xie et al., Direct ink writing of adjustable electrochemical energy storage device with high gravimetric energy densities. Adv. Funct. Mater. **29**, 1900809 (2019). https://doi.org/10.1002/adfm.201900809

[6] T.T. Gao, Z. Zhou, J.Y. Yu, J. Zhao, G.L. Wang et al., 3D printing of tunable energy storage devices with both high areal and volumetric energy densities. Adv. Energy Mater. **8**, 1802578 (2018). https://doi.org/10.1002/aenm.201802578

[7] M.Y. Zhao, Y.L. Li, F. Lin, Y.S. Xu, L.L. Chen et al., A quasi-solid-state photothermal supercapacitor via enhanced solar energy harvest. J. Mater. Chem. A **8**, 1829-1836 (2020). https://doi.org/10.1039/C9TA11793H

[8] H.Y. Liang, J.H. Lin, H.N. Jia, S.L. Chen, J.L. Qi et al., Hierarchical NiCo-LDH@NiOOH core-shell heterostructure on carbon fiber cloth as battery-like electrode for supercapacitor. J. Power Sources **378**, 248-254 (2018). https://doi.org/10.1016/j.jpowsour.2017.12.046

[9] B. Anothumakkoolab, R. Soniab, S.N. Bhangea, S. Kurungotab, Novel scalable synthesis of highly conducting and robust PEDOT paper for a high performance flexible solid supercapacitor. Energy Environ. Sci. **8**, 1339-1347 (2015). https://doi.org/10.1039/C5EE00142K

[10] C.R. Zhu, P.H. Yang, D.L. Chao, X.L. Wang, X. Zhang et al., All Metal Nitrides Solid ‐ State Asymmetric Supercapacitors. Adv. Mater. **27**, 4566-4571 (2015). https://doi.org/10.1002/adma.201501838

[11] X. Xiao, X. Peng, H.Y. Jin, T.Q. Li, C.C. Zhang et al., Freestanding mesoporous VN/CNT hybrid electrodes for flexible all-solid-state supercapacitors. Adv. Mater. **25**, 5091-5097 (2013). https://doi.org/10.1002/adma.201301465

[12] B. Yao, L.Y. Yuan, X. Xiao, J. Zhang, Y.Y. Qi et al., Paper-based solid-state supercapacitors with pencil-drawing graphite/polyaniline networks hybrid electrodes. Nano Energy **2**, 1071-1078 (2013). https://doi.org/10.1016/j.nanoen.2013.09.002

[13] H. Wu, K. Jiang, S.S. Gu, H. Yang, Z. Lou, D. Chen, G.Z. Shen, Two-dimensional Ni(OH)2 nanoplates for flexible on-chip microsupercapacitors. Nano Res. **8**, 3544-3552 (2015). https://doi.org/10.1007/s12274-015-0854-3

[14] Z.W. Peng, R.Q. Ye, J.A. Mann, D. Zakhidov, Y.L. Li et al., Flexible Boron-doped laser-induced graphene microsupercapacitors. ACS Nano **6**, 5868−5875 (2015). https://doi.org/10.1021/acsnano.5b00436

[15] S.K. Kim, H.J. Koo, A. Lee, P.V. Braun, Selective wetting-induced micro-electrode patterning for flexible micro-supercapacitors. Adv. Mater. **26**, 5108-5112, (2014) https://doi.org/10.1002/adma.201401525

[16] L.H. Yu, Z.D. Fan, Y.L. Shao, Z.N. Tian, J.Y. Sun, Z.F. Liu, Versatile N-doped MXene ink for printed electrochemical energy storage application. Adv. Energy Mater. **9**, 1901839 (2019). https://doi.org/10.1002/aenm.201901839

[17] X. Xiao, T.Q. Li, P.H. Yang, Y. Gao, H.Y. Jin et al., Fiber-based all-solid-state flexible supercapacitors for self-powered systems ACS Nano **6**, 9200-9206 (2012). https://doi.org/10.1021/nn303530k

[18] Y.Y. Wanga, Y.Z. Zhang, D. Dubbinka, J.E. Elshofa, Inkjet printing of δ-MnO_2 nanosheets for flexible solid-state micro-supercapacitor. Nano Energy **49**, 481-488 (2018). https://doi.org/10.1016/j.nanoen.2018.05.002

[19] H. Pang, Y.Z. Zhang, W.Y.Lai, Z. Huc, W. Huang, Lamellar $K_2Co_3(P_2O_7)_2{\cdot}2H_2O$ nanocrystal whiskers: High-performance flexible all-solid-state asymmetric micro-supercapacitors via inkjet printing. Nano Energy **15**, 303-312 (2015). https://doi.org/10.1016/j.nanoen.2015.04.034
